# Gut Structure and Microbial Communities in *Sirex noctilio* (Hymenoptera: Siricidae) and Their Predicted Contribution to Larval Nutrition

**DOI:** 10.3389/fmicb.2021.641141

**Published:** 2021-04-08

**Authors:** Jiale Li, Chengcheng Li, Ming Wang, Lixiang Wang, Xiaobo Liu, Chenglong Gao, Lili Ren, Youqing Luo

**Affiliations:** ^1^Beijing Key Laboratory for Forest Pest Control, Beijing Forestry University, Beijing, China; ^2^Sino-France Joint Laboratory for Invasive Forest Pests in Eurasia, Beijing Forestry University, Beijing, China

**Keywords:** gut microbiota, gut structure, larval nutrition, lignocellulose, nitrogen fixation, pest, *Sirex noctilio*

## Abstract

The European woodwasp, *Sirex noctilio* Fabricius, is a major invasive quarantine pest that attacks and kills pine trees outside of its native range. Insect gut structure and gut microbiota play crucial roles in various life activities. Despite a few reports in nutrition and survival, an extensive study on the *S. noctilio* larval gut microbiome is lacking. We studied the gut structure using a stereo microscope and used high throughput sequencing of the bacterial 16S rRNA genes and fungal internal transcribed spacer 2 (ITS2) regions to investigate gut microbiota in different developmental stages of *S. noctilio*, including larvae, adults, and larval frass. We used PICRUSt2 to predict the functional profiles. The larval gut was thin and thread-like from the oral cavity to the anus, carrying few xylem particles in the crop. *Pseudomonas*, *Ralstonia*, and *Burkholderia* s.l were the dominant bacteria in the guts of larvae, adults, and frass, respectively. Even though *Pseudomonas* was the most abundant among all bacteria, *Zoogloea*, *Ruminobacter*, and *Nitrosospira*, which might be involved in degrading organic matter and fixing nitrogen occurred exclusively in the larval gut indicating their possible role in the growth and development of larvae in pine tree xylem. Fungal communities did not change significantly across different developmental stages or the frass. *Amylostereum* was dominant in the woodwasp’s larval gut. Functional prediction of bacterial and fungal communities revealed that they may encod enzymes involved in degrading lignocellulose and fixing nitrogen. Ours is the first study that compares gut microbial communities present in *S. noctilio* larvae, adults, and frass. This study could provide an understanding of larval nutrient acquisition in nutrient-deficient host xylem to some extent. Our study may unlock novel strategies for the development of pest management approaches based on interfering with the gut microbiota and restricting their role in larval survival and development.

## Introduction

Insects are the most abundant animals on earth, inhabiting diverse habitats and feeding on various substrates (Basset et al., 2012). Insects are colonized by different microorganisms including bacteria, fungi, protozoa, and archaea, which are often beneficial to their hosts (Ferrari and Vavre, 2011; Douglas, 2015). This association is mutually beneficial as insect guts facilitate the growth of microorganisms, and the microorganisms in the gut, in turn, provide support to their hosts in nutrition, digestion, development, reproduction, defense, behavior, and survival (Hammer and Bowers, 2015; Prasad et al., 2018). Recent years, with the developing of molecular biology techniques, research on the gut microbiota and biological functions of insects have shown potential implication in pest control (Whitten et al., 2016). To fully understand insects’ life cycle, it is imperative to consider the role of microorganisms inhabiting the insects.

The pine xylem, made up of refractory lignocellulosic bonds, has low nutrient contents and lacks certain amino acids and sterols essential for protein synthesis (Mattson, 1980; Geib et al., 2008). Many insects can adapt to a range of ecological niches, where they often thrive on nutrient-poor or refractory diets through various morphological and physiological adaptations (Chiappini and Aldini, 2011; Philipp and Moran, 2013). For example, some ant species have several specialized gut bacteria and associated morphological modifications of the gut (Caetano et al., 2009; Russell et al., 2009). In different ant genera, herbivory is strongly correlated with the prevalence of Rhizobiales as gut symbionts (Russell et al., 2009). Moreover, gut bacteria in termites can either utilize nitrogenous waste products excreted by the host and recycle them into nutrients or directly fix nitrogen from the atmosphere (Hongoh et al., 2008; Thong-On et al., 2012).

The European woodwasp, *Sirex noctilio* Fabricius, is a major invasive quarantine pest that damages *Pinus* species in its invaded areas and impacts the economy (Foelker, 2016). It is the only known woodwasp species that could kill living trees by overcoming their self-defenses (Spradbery, 1973). Li et al. (2015) discover and identify *S*. *noctilio* in Heilongjiang Province, indicating that this invasive pest has invaded China. So far, 22 regions in China have been invaded by *S. noctilio*, thereby endangering Mongolian pine plantations (Sun et al., 2016; Wang et al., 2019). Based on the CLIMEX model, researchers predict that *S. noctilio* could colonize most of the areas from Yunnan Province to Heilongjiang Province in China, where many susceptible hosts are planted (Ireland et al., 2018). Li et al. (2019) conclude that there is a high risk that *S. noctilio* may bring great damages to China’s pine forests.

*Sirex noctilio*, which develops in pine trees’ xylem, lives its larval stage sealed inside the wood (Thompson, 2013). Some research have been done on how *S. noctilio* obtains sufficient nutrients in pine woods to survive, and it has special organs call “mycangia” and “venom glands” inside its female abdomen. The female adult infects the host as a complex damage system with its venom mucus in the body and its symbiotic fungus *Amylostereum areolatum* (Coutts, 1969; Ryan and Hurley, 2012). The venom glands secrete venom mucus and store them in a mucus reservoir, those organs are all connected with the female’s ovipositor (Madden, 1974). Hence, *S. noctilio* is not only a wood-borer pest, but also injects its symbiotic fungus *A. areolatum* and toxins (secreted by its venom gland) into the host tree when the female lays eggs (Ryan and Hurley, 2012). It is a cooperative damage mechanism working together to weak the hosts. What’s more, the larva cannot produce sufficient lignocellulosic enzymes or feed directly on the host xylem, so *A. areolatum* helps it as “external rumen” for larval absorption (Madden, 1981; Thompson et al., 2013, 2014). Laccase enzyme activity is identified from *A. areolatum* (Bordeaux, 2008). Laccase is a lignin-degrading enzyme belonging to the AA1 family. Fu et al. (2020) detect 62 and 25 copies of AA3 and AA1 in *A. areolatum*, respectively.

Despite several nutrition and survival reports, an extensive study on the *S. noctilio* larval gut microbiome is lacking. We hypothesized that the gut microbiota must play an imperative role in the development and survival of *S*. *noctilio* in the nutrient-deficient host xylem. To test this hypothesis, we studied the gut structure and explored high throughput sequencing of the bacterial 16S rRNA genes and fungal internal transcribed spacer 2 (ITS2) region to investigate gut microbial communities throughout the ontogeny of *S. noctilio*, including larvae, female and male adults, and larval frass.

## Materials and Methods

### Sample Collection and Larval Identification

The larvae and adults of *S. noctilio* were collected from northeastern China, Duerbert Mongolian Autonomous County, Daqing City, Heilongjiang Province, China, from April to August 2019 (46.88°N, 124.46°E). We randomly selected *P. sylvestris* var. *mongolica* (the stem of trees had drilled holes and resin drops; Madden, 1974), cut sample pines into wood logs, and brought them back to the quarantine laboratory (Beijing Forestry University). The larvae and frass collection work were carried out using a wood splitter (LS7T-520, Baiduan Industry and Trade Co., Ltd., Shanghai, China) and axe. Each sample was immediately placed in a clean and sterilized 1.5-mL centrifuge tube and temporarily stored at −80°C before use in the experiments. Since larval instars were unknown, we used the body size to determine the relative age of the larvae (body length is about 1.0–1.5 cm), avoiding collecting very young larvae or older larvae (pre-pupae). The adults were collected in the quarantine laboratory during the whole emergence period, and the subsequent collection work was similar to that of larvae.

Since the larval species was difficult to identify based on morphology, we identified the species by extracting the DNA from the collected larval heads. The PCR amplicons were obtained by using LCO1490 and HCO2198 primers (Folmer et al., 1994) targeting *cytochrome c oxidase (COI) subunit I* gene (Supplementary Table 1). BLAST was performed on the NCBI website to confirm whether it was *S. noctilio.*

### Gut Dissection and Observation

We prepared 75% (v/v) ethanol, sterile water, and phosphate-buffered saline (PBS) solution before dissection in PBS solution. The work area and instruments were surface-sterilized with 75% (v/v) ethanol. Larvae and adults were fixed on the wax plate and carefully dissected using a dissection microscope under aseptic conditions. A pair of micro-scissors was used to cut from the end of the abdomen to the head. The gut was picked gently and placed in sterile water to wash any attached fat body or tissue. The guts were then placed in a pre-sterilized 1.5-mL centrifuge tube and immediately flash-frozen with liquid nitrogen. The samples were stored at −80°C until processed for DNA extraction. The larval guts were observed by a stereoscopic microscope (Leica M205FA, United States), and then the parts of the gut were described in detail.

### Total DNA Extraction, PCR Amplification, and Sequencing

Total DNA was extracted from gut samples (entire digestive tracts from five individuals; five guts/frass for one sample; 5–7 replicates of pools per life stage and frass). All samples (five replicates larvae gut, six replicates female adult gut, six replicates frass, and seven replicates male adult gut, respectively) were ground in liquid nitrogen using sterile pestles. The ZR Fecal and Soil DNA MicroPrep (Epigenetics, United States) was used for DNA extraction following the manufacturer’s protocol. The total DNA concentration and quality were estimated with a NanoDrop 2000 spectrophotometer (Thermo Fisher Scientific, Wilmington, DE, United States) and an Agilent 2,100 Bioanalyzer (Agilent Technologies, Palo Alto, CA, United States), respectively. Gel electrophoresis was used to assess the integrity of the total DNA in the samples.

The PCR reactions were carried out in 20 μL of a solution containing 10 ng DNA, 5 μM each primer 0.8 μL, BSA 0.2 μL, 2.5 mM dNTPs 2 μL, Fastpfu polymerase 0.4 μL (Transgene, China), 5× Fastpfu buffer 4 μL, and supplemented ddH_2_O to 20 μL. Forward primer 338F and reverse primer 806R (Xu et al., 2016) were used to amplify V3 to V4 variable regions of the bacterial 16S rRNA gene; forward primer ITS3F and reverse primer ITS4R (Toju et al., 2012) were employed to amplify the fungal ITS2 region (Supplementary Table 1). The amplification was performed in an ABI GeneAmp 9,700 thermal cycler under the following conditions: 95°C for 3 min, followed by 27 cycles consisting of denaturation at 95°C for 30 s, annealing at 55°C for 30 s, extension at 72°C for 45 s, and the final extension step at 72°C for 10 min. We included appropriate negative controls at the DNA extraction and all steps in PCR reactions. The final PCR products were analyzed by electrophoresis in 2% (w/v) agarose gel followed by staining with Gelred and visualization under ultraviolet light. The 16S rRNA and ITS2 region were amplified in triplicates and mixed with DNA. Equal volumes were pooled for Illumina MiSeq sequencing (PE300 platform) according to the standard protocol by Majorbio Bio-Pharm Technology Co., Ltd. (Shanghai, China).

### Sequence Data Processing

Raw sequences were deduplicated and quality-filtered by fastp version 0.20.0 (Chen et al., 2018). Paired-end reads were merged using FLASH v1.2.7 (Magoč and Salzberg, 2011) with the following criteria: (i) the 300 bp reads were truncated at any site receiving an average quality score of <20 over a 50 bp sliding window, and the truncated reads shorter than 50 bp were discarded, reads containing ambiguous characters were also discarded; (ii) only overlapping sequences longer than 10 bp were assembled according to their overlapped sequence. The maximum mismatch ratio of the overlap region is 0.2. Reads that could not be assembled were discarded; (iii) Samples were distinguished according to the barcode and primers. The sequence direction was adjusted, exact barcode matching, two nucleotide mismatches in primer matching. After that, all reads from each sample were clustered into operational taxonomic units (OTUs) at a 97% sequence similarity cutoff using the Uparse pipeline of Usearch v7.1^1^ (Edgar, 2013). A representative sequence was selected from each OTU using default parameters, and then taxonomic classification was conducted using the RDP Classifier v2.2^2^ (Wang et al., 2007). Bacterial reads were compared to the SILVA 138 database (Quast et al., 2012) using a confidence threshold of 70%, while, for fungal reads, the UNITE v8.0 database was used (Nilsson et al., 2019). Taxonomies were grouped at the phylum, class, order, family, and genus levels. OTUs identified as unclassified bacteria or fungi at the phylum level, archaeans, mitochondria, or chloroplasts were excluded. These were classified as additional quality control or contaminants, and removed before analysis. OTUs that were < 1% of average relative abundance in groups were summarized as “others.”

### Statistical Analysis

The USEARCH generated OTU tables were modified into a shared compatible file and then uploaded into Mothur v1.30.2 (Schloss et al., 2009) to make rarefaction curves to estimate species richness and diversity. Alpha diversity analysis was calculated for the different woodwasps groups using Mothur v1.30.2^3^ and visualized using R packages “vegan.” The Shannon, Simpson, ACE, Chao, and coverage indices were calculated. Among this, Chao and Shannon indices were calculated for the different groups, and then the Wilcoxon rank-sum test was used to calculate significant differences (^∗^0.01 < *P* ≤ 0.05, ^∗∗^0.001 < *P* ≤ 0.01). Beta diversity analyses were conducted using QIIME v1.9.1 (Caporaso et al., 2010) and visualized using R packages “vegan” and “ggplot2” in R v3.6.1. Beta diversity analysis was performed to investigate structural variation in microbial communities of the different group samples using Unweighted and Weighted UniFrac distance metrics (Lozupone and Knight, 2005) principal coordinates analysis (PCoA). The significance of differentiation of microbiota structure among groups was assessed by adonis or permutational multivariate analysis of variance (PERMANOVA) (Anderson, 2001) with 999 permutations.

Bacterial and fungal taxa analyses were plotted using the R package “barplot”. Based on the modified OTUs data, the difference in the top ten genera relative abundance between different groups of microbial community species was compared by one-way ANOVA, followed by Scheffe test (^∗^0.01 < *P* ≤ 0.05, ^∗∗∗^*P* ≤ 0.001), and was conducted using the R package “stats”. Correlation analysis was carried out by R package “ggplot2” and “corrplot” (Significant correlations are shown as ^∗^*P* < 0.05, ^∗∗^*P* < 0.01, and ^∗∗∗^*P* < 0.001).

Microbial community functions were predicted by phylogenetic investigation of communities by reconstruction of unobserved states v1.1.0 (PICRUSt2 v1.1.0) (Langille et al., 2013) based on high-quality sequences. The PICRUSt2 generates predictions from 16S rRNA and ITS2 data using annotations of sequenced genomes in the Greengene database and KEGG database release 64.0.

## Results

### Gut Structure of *S. noctilio* Larva

**FIGURE 1:**
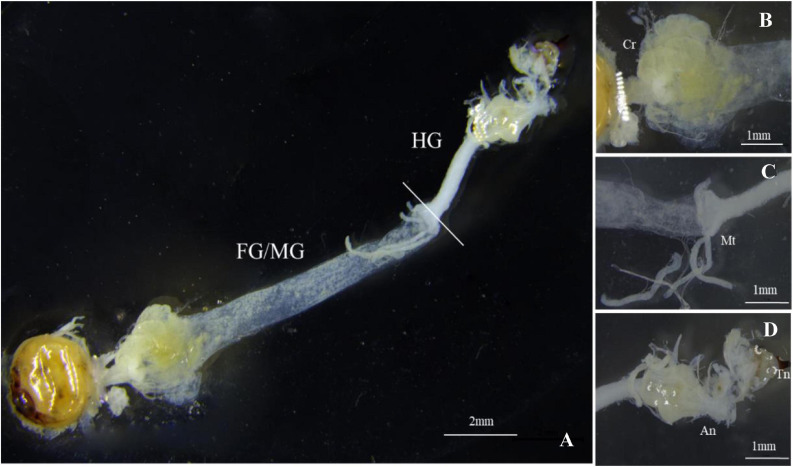
Gut morphology of *Sirex noctilio* larva. **(A)** The digestive tract includes foregut (FG), midgut (MG), and hindgut (HG). Enlarged images of **(B)** Crop (Cr), **(C)** Malpighian tubules (Mt), and **(D)** Anus (An) and Tail needle (Tn). Photographs were taken using Leica M205FA, United States.

The gut structure of *S. noctilio* was relatively simple. It accounted for the largest proportion of the body cavity except for the fat body. The gut was thin and thread-like from the oral cavity to the anus. It consisted of three regions: foregut, midgut, and hindgut, which lacked distinct fermentation chambers found in the typical gut structure of xylophagous insects. We observed few xylem particles in the crop of foregut. Foregut and midgut were not separated, and they represented the majority of the digestive tract. Malpighian tubules branched from the midgut-hindgut border (Figures 1A–D). In addition, the fat body of the larva filled most of the body cavity and enveloped the reticulate salivary glands.

### NGS Sequencing

A total of 1,088,965 paired-end reads of 16S rRNA V3–V4 amplicon sequences and 1,167,000 reads of ITS amplicon sequences were generated to survey the bacterial and fungal communities, respectively. After quality filtering, we obtained 663,494/895,353 high-quality sequences (an average of 27,646/37,306 reads per sample), from which 1,902/293 OTUs were identified to be from 24 samples (Supplementary Tables 2,4). The OTU-level rarefaction curves were generated to compare the richness and evenness of OTUs among samples (Supplementary Figures 1,2), indicating that these specimens’ sequencing depths were appropriate. Detected OTU numbers, Shannon, Simpson, ACE, Chao, and coverage indices were estimated as alpha diversity indicators (Supplementary Table 3).

### Bacterial Communities Associated With the Larvae and Adult Guts, and Frass of *S. noctilio*

**FIGURE 2:**
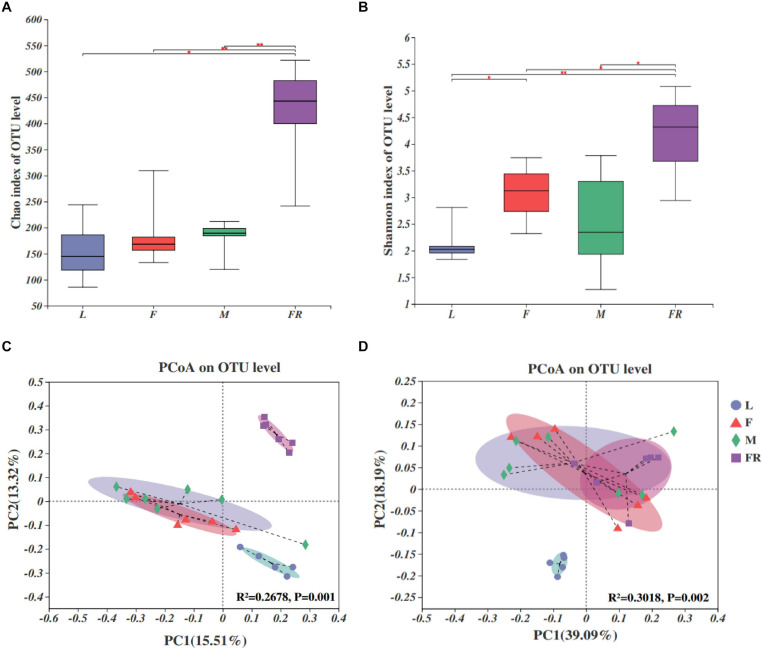
Bacterial communities in the *Sirex noctilio* gut and frass. Boxplots of **(A)** species richness (Chao index), and **(B)** species diversity (Shannon index). The significant differences of alpha diversities were analyzed using Wilcoxon rank-sum test (^∗^0.01 < *P* ≤ 0.05, ^∗∗^0.001 < *P* ≤ 0.01). **(C)** Unweighted and **(D)** weighted UniFrac-based PCoA plots of bacterial communities. The significant differences in beta diversities were analyzed using adonis analysis with 999 Monte Carlo permutations (L, larva; F, female; M, male; and FR, frass).

Alpha diversity analyses showed that bacterial species richness (observed OTUs, Chao index) and species diversity (Shannon index) were both significantly different among larvae, adults, and frass (Figures 2A,B). The trends of the Chao index and Shannon index were similar. Alpha diversity was significantly higher in frass samples compared with adult samples and larva samples. The alpha diversity value was the highest in frass, followed by adults, and the lowest in larvae (Figures 2A,B). The results showed no significant difference in the alpha diversity of gut microbiota between the female and male adults, and between the larvae and adults. The Shannon index of gut microbiota in larvae was significantly lower than in females (*P* = 0.022) (Figure 2B).

Comparison of different groups of microbiota using PCoA based on unweighted and weighted UniFrac metric data, showed distinct clustering, with most of the variation explained by the first two coordinates (Figures 2C,D, *R*^2^ = 0.2678, and *P* = 0.001 for unweighted; *R*^2^ = 0.3018 and *P* = 0.002 for weighted, adonis**)**. Individuals from larva and frass were clustered separately, whilst individuals in frass were clustered together with individuals in female adults and male adults based on weighted UniFrac metric data (Figure 2D). Most individuals from female and male adults were clustered together. The results of PERMANOVA showed significant differences between the two groups (*P* ≤ 0.001) (Supplementary Table 5). Our findings indicated that bacterial communities changed significantly with the growth and development of *S. noctilio*, and the larval gut communities differed markedly from those present in adults or frass.

**FIGURE 3:**
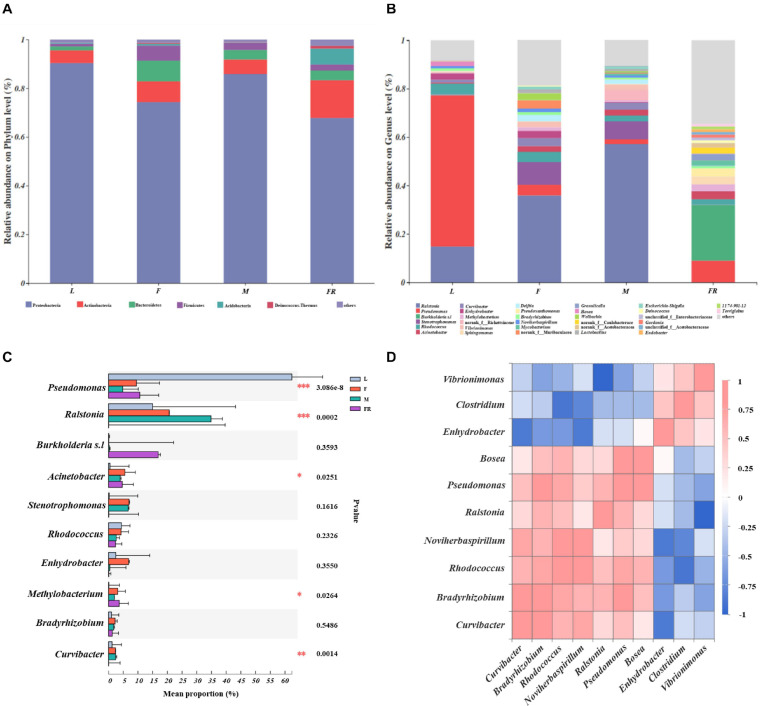
Taxonomic composition of bacterial communities associated with *Sirex noctilio* gut and frass. Relative abundance of each bacterial **(A)** phylum, and **(B)** genus. Each bar is indicated by a different color at phylum and genus level. OTUs that were <1% of average relative abundance in groups are summarized as “others”. **(C)** Significant differences of microbial composition in larvae, female adults, male adults, and frass in the relative abundance of top ten (one-way ANOVA followed by Scheffe test; ^∗^0.01 < *P* ≤ 0.05, ^∗∗^0.001 < *P* ≤ 0.01, ^∗∗∗^*P* ≤ 0.001). **(D)** Spearman correlations of samples with the OTU abundance of genus in *S. noctilio* larval gut in the relative abundance of top ten. Blue boxes represent co-exclusion/negative correlations; red boxes represent co-occurrence/positive correlations between microbes (L, larva; F, female; M, male; and FR, frass).

The relative abundance of bacterial communities among different groups of *S. noctilio* was examined at phylum and genus levels (Figures 3A,D and Supplementary Figure 3). The taxonomic analysis at the phylum level revealed that the bacterial communities inhabiting *S. noctilio* were predominantly Proteobacteria, followed by Actinobacteria, Bacteroidetes, Firmicutes, Acidobacteria, and Deinococcus-Thermus (Figure 3A). At the genus level, the dominant groups were *Ralstonia*, *Pseudomonas*, *Burkholderia* s.l, *Curvibacter*, *Acinetobacter*, *Enhydrobacter*, *Methylobacterium*, *Bradyrhizobium*, *Bosea*, *Stenotrophomonas*, and *Pseudoxanthomonas* of phylum Proteobacteria; *Rhodococcus*, *Mycobacterium*, and *Microbacterium* of phylum Actinobacteria; *Vibrionimonas* and *Hydrotalea* of phylum Bacteroidetes; *Lactobacillus* and *Staphylococcus* of phylum Firmicutes; *Granulicella* and *Bryobacter* of phylum Acidobacteria; *Deinococcus* of phylum Deinococcus-Thermus (Figure 3B).

We observed that each group had its own significantly enriched set of microorganisms at the genus level (excluding unclassified genera) (Figure 3C). For instance, *Pseudomonas* was notably enriched in the larval gut as compared to other groups (*P* ≤ 0.001), whereas *Ralstonia* was the most abundant bacteria in the male gut (*P* ≤ 0.001). *Acinetobacter* was notably enriched in the gut of female adults, whilst *Methylobacterium* was significantly enriched in frass. The shared groups among different groups of *S. noctilio* were shown in Venn diagrams, a total of 183 OTUs (9.62%) and 79 genera (10.93%) were only present in larval groups (Supplementary Figure 4, see details Supplementary Table 6). The common genera suggested that they might have essential functions in the growth and development of *S. noctilio*.

To understand the co-occurrence pattern of *S. noctilio* larval gut bacterial communities, a heatmap correlation analysis was established based on significant correlations among different bacteria for the top ten genera (Figure 3D, Spearman, *r* ≥ 0.5, *P* < 0.05). The high abundance bacterium *Pseudomonas* exhibited co-occurrence correlations with *Bradyrhizobium* (*r* = 0.8), *Rhodococcus* (*r* = 0.7), *Ralstonia* (*r* = 0.6), and *Bosea* (*r* = 0.9), whereas it showed negative interactions with *Vibrionimonas* (*r* = −0.6). Similarly, the high abundance bacterium *Ralstonia* had negative interactions with *Vibrionimonas*. The highly abundant bacteria *Rhodococcus* and *Bradyrhizobium* showed co-occurrence correlations (*r* = 0.7). The two genera had similar correlations with *Curvibacter, Noviherbaspirillum, Ralstonia, Pseudomonas*, and *Bosea*, but had negative interactions with *Enhydrobacter*, *Vibrionimonas*, and *Clostridium*.

### Fungal Communities Associated With the Larvae and Adult Guts, and Frass of *S. noctilio*

**FIGURE 4:**
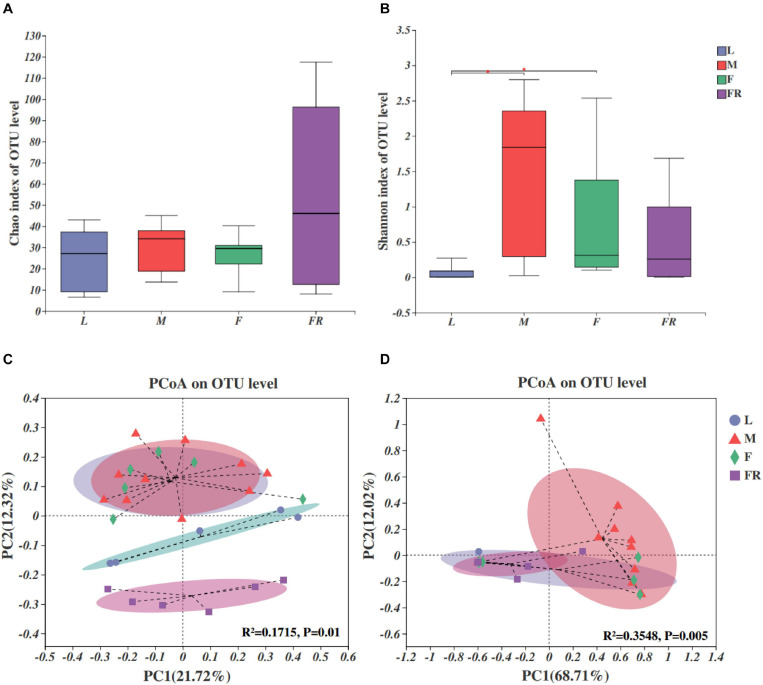
Fungal communities in the *Sirex noctilio* gut and frass. Boxplots of **(A)** species richness (Chao index), and **(B)** species diversity (Shannon index). The significant differences of alpha diversities were analyzed using Wilcoxon rank-sum test (^∗^0.01 < *P* ≤ 0.05). **(C)** Unweighted and **(D)** weighted UniFrac-based PCoA plots of fungal communities. The significant differences in beta diversities were analyzed using adonis analysis with 999 Monte Carlo permutations (L, larva; F, female; M, male; and FR, frass).

The fungal compositions were simpler as compared with those of bacteria in *S. noctilio* (Figures 4A,B). Both the numbers of observed OTUs and Shannon index were less for fungi than bacteria. Alpha diversity analyses showed that fungal species richness (observed OTUs, Chao index) and species diversity (Shannon index) were not significantly different among different groups. However, we found that the Shannon index of gut microbiota in larvae was significantly lower than those in males (*P* = 0.012) and females (*P* = 0.035) (Figure 4B).

Comparison of different groups of microbiota, using PCoA based on unweighted and weighted UniFrac metric data, showed distinct clustering, with most of the variation explained by the first two coordinates (Figures 4C,D, *R*^2^ = 0.1715, and *P* = 0.01 for unweighted; *R*^2^ = 0.3548 and *P* = 0.005 for weighted, adonis**)**. The fungal community structure was roughly similar between female and male adults in either unweighted or weighted UniFrac analysis. They harbored more significant numbers and more diverse fungi than other groups (larva and frass). Individuals from larva and frass groups clustered separately for unweighted UniFrac analysis (Figure 4C), and together for weighted UniFrac analysis (Figure 4D). The results of PERMANOVA showed significant differences between the two groups (*P* ≤ 0.001) (Supplementary Table 7). Our findings indicated that the fungal community’s composition did not change with the growth and development of *S. noctilio*, and the adult gut communities were more abundant.

**FIGURE 5:**
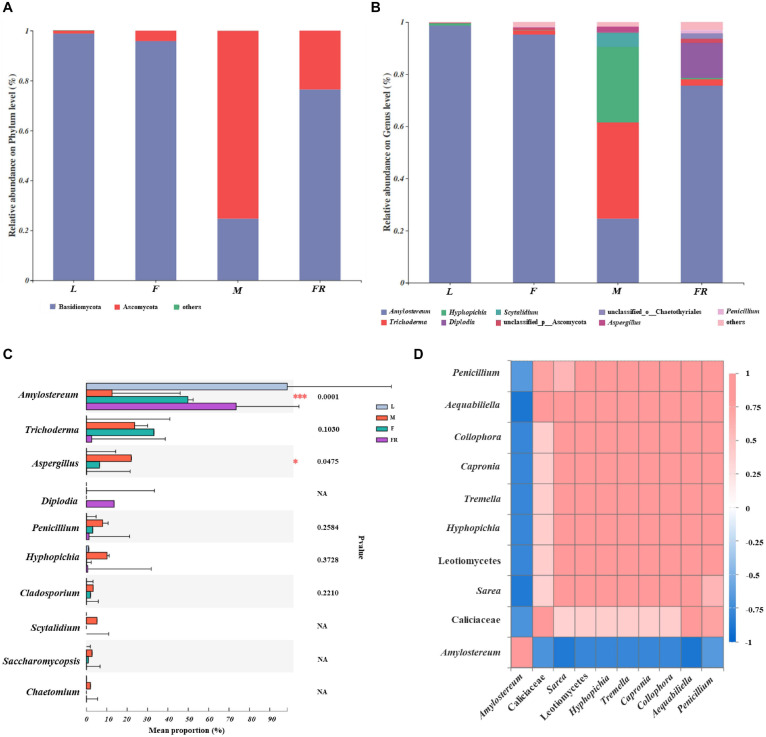
Taxonomic composition of fungal communities associated with *Sirex noctilio* gut and frass. Relative abundance of each fungal phylum and genus **(A,B)**. Each bar is indicated by a different color at phylum and genus level. OTUs that were <1% of average relative abundance in groups are summarized as “others”. **(C)** Significant differences of microbial composition in larvae, female adults, male adults and frass in the relative abundance of top ten (one-way ANOVA followed by Scheffe test, ^∗^0.01 < *P* ≤ 0.05, ^∗∗∗^*P* ≤ 0.001). **(D)** Spearman correlations of samples with the OTU abundance of genus in *S. noctilio* larval gut in the relative abundance of top ten. Bule boxes represent co-exclusion/negative correlations; red boxes represent co-occurrence/positive correlations between microbes (L, larva; F, female; M, male; and FR, frass).

The relative abundance of fungal communities among different *S. noctilio* groups was examined at phylum and genus levels (Figure 5 and Supplementary Figure 5). Basidiomycota and Ascomycota mainly dominated the fungal communities in relation to *S. noctilio* at the phylum level (Figure 5A). The phylum Basidiomycota was represented primarily by *Amylostereum*, *Tremella*, and *Malassezia* at the genus level. The phylum of Ascomycota was represented primarily by *Trichoderma, Simplicillium*, *Hyphopichia*, *Diplodia*, *Scytalidium*, *Aspergillus*, and *Penicillium* (Figure 5B).

The top ten fungal genera (excluding unclassified genera) inhabiting in larvae, adults, and frass of *S. noctilio* also varied (Figure 5C). For example, *Amylostereum* was notably enriched in the larval gut as compared with other groups (*P* ≤ 0.001), whereas *Aspergillus* was the most abundant fungus in the male gut (*P* ≤ 0.05). The shared groups among four groups of *S. noctilio* were shown in Venn diagrams, a total of 9 OTUs (3.07%) and seven genera (4.35%) only were present in the larval group (Supplementary Figure 6, see details Supplementary Table 8). The common genera suggested that they might have important functions in the growth and development of *S. noctilio*, especially *Amylostereum*.

A heatmap correlation analysis was established based on significant correlations among different fungi for the top ten genera in *S. noctilio* larva gut (Figure 5D, Spearman, *r* ≥ 0.5, *P* < 0.05). The high abundance fungus *Amylostereum* had negative interactions with other fungal genera to varying degrees, which explained, to some extent, why it was present abundantly. Meanwhile, the high abundant fungus “g_unclassified_f_Callciaceae” had slight co-occurrence correlations with other fungal genera. Conversely, except for the above two, the remaining fungal genera showed a strong positive correlation.

### Co-Occurrence of Bacteria and Fungi in *S. noctilio* Larval Gut

**FIGURE 6:**
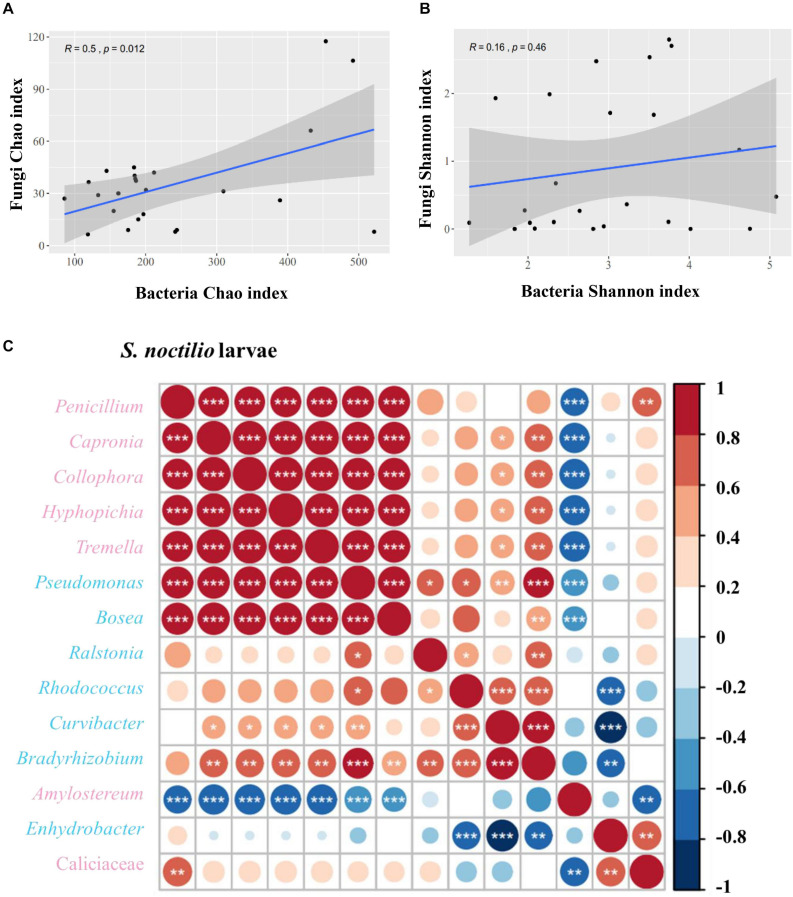
Correlation of bacterial and fungal alpha diversity indices in *Sirex noctilio* larval gut. **(A)** Chao index and **(B)** Shannon index. The Pearson correlation coefficient (*R*) and the significance level (*P*-value) are shown on the plots. **(C)** Spearman correlations of samples with the OTU abundance of bacterial and top seven fungal genera in *S. noctilio* larval gut. Positive correlations are displayed in red and negative correlations in blue. Color intensity and the size of the circles are proportional to the correlation coefficients. Significant correlations are shown as *P < 0.05, **P < 0.01, and ***P < 0.001. The pink font indicates fungal genera and light blue font indicates bacterial genera.

We determined the correlations between bacteria and fungi within *S. noctilio* larval gut, and found the species richness (Chao index) of bacteria within the community were significantly correlated to those of fungi (*R* = 0.5, *p* = 0.012) (Figure 6A), whilst the species diversity (Shannon index) was not significantly correlated (*R* = 0.16, *p* = 0.46) (Figure 6B). In addition, most of the highly abundant bacterial and fungal genera were positively correlated to varying degrees (Figure 6C). However, the relative abundance of *Amylostereum* was negatively correlated to almost all other bacterial and fungal genera, and *Enhydrobacter* also showed a slightly similar trend.

### Microbial Functions Predicted via PICRUSt2 in *S. noctilio* Larval Gut

**FIGURE 7:**
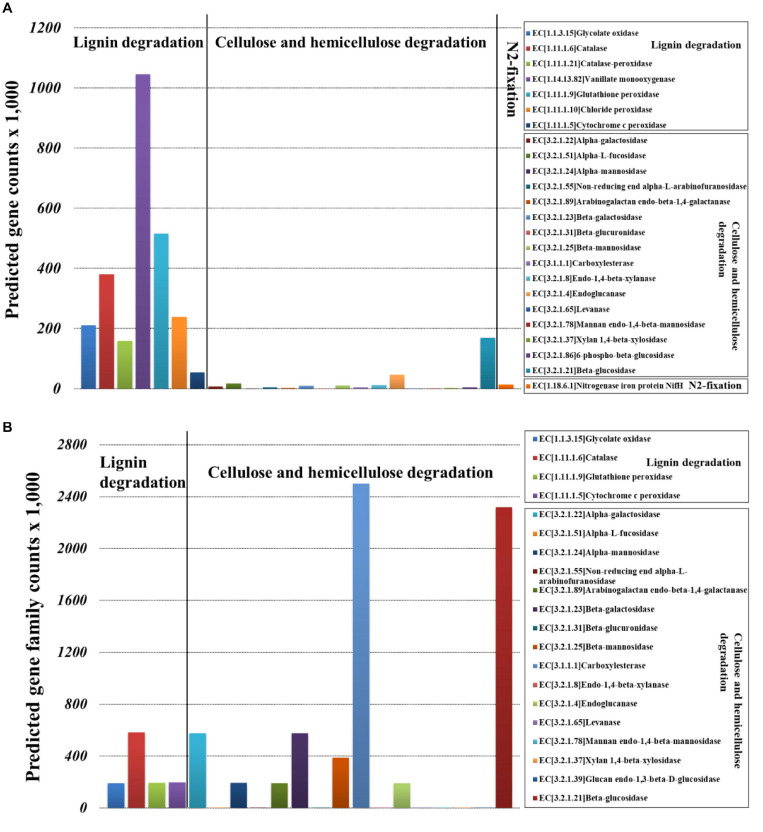
Selection of genes involved in the lignocellulose degradation and nitrogen fixation of *Sirex noctilio* larval gut. **(A)** Bacterial and **(B)** fungal communities (the sum number of predicted gene family counts).

Bacterial community functional prediction showed 23 genes potentially related to lignocellulose degradation and one gene potentially related to nitrogen fixation (Figure 7A), and the detailed enzyme-catalyzed reactions were demonstrated in Supplementary Table 9. Based on the lignocellulose degradation pathways, most of the predicted genes were involved in lignin degradation, including vanillate monooxygenase (EC1.14.13.82), glutathione peroxidase (EC1.11.1.9), catalase (EC1.11.1.6), chloride peroxidase (EC1.11.1.10), glycolate oxidase (EC1.1.3.15), catalase-peroxidase (EC1.11.1.21), and cytochrome c peroxidase (EC1.11.1.5). For cellulose and hemicellulose degradation, beta-glucosidase (EC3.2.1.21) was the most abundant. For biological nitrogen fixation, we predicted key genes for nitrogenase component proteins (nifH).

Fungal community functional prediction showed 20 genes potentially related to lignocellulose degradation and no gene potentially related to nitrogen fixation (Figure 7B). The detailed enzyme-catalyzed reactions were demonstrated in Supplementary Table 10. Based on the lignocellulose deconstruction pathways, most of the predicted genes were involved in cellulose and hemicellulose degradation, including beta-glucosidase (EC3.2.1.21), carboxylesterase (EC3.1.1.1), alpha-galactosidase (EC3.2.1.22), beta-galactosidase (EC3.2.1.23), and beta-mannosidase (EC3.2.1.25). For lignin degradation, catalase (EC1.11.1.6) was the most abundant.

## Discussion

### Contribution of Gut Structure to *S. noctilio* Larval Nutrition

The structure of *S. noctilio* larval gut was simple; it was thin and thread-like from the oral cavity to the anus, without any specialized structures. We observed that foregut and midgut were not clearly separated, few xylem particles were present in the crop that might temporarily store food for subsequent digestion. Bignell (1981) also report the digestion of cellulose and hemicellulose in the crop of cockroaches using enzymes from the midgut, which was colonized by bacteria. Some stinkbugs have specialized midgut crypts that provide places for microbes to colonize (Bistolas et al., 2014). Otherwise, this is different from other wood-feeding insects, such as higher termites, and the hindgut of them has specialized structure called fermentation chamber that can harbor endosymbiotic microorganism to help in wood digestion (Warnecke et al., 2007). Some wood-feeding insects’ hindgut microorganisms can even use the uric acid released by the Malpighian tubules as a nitrogen source (Philipp and Moran, 2013). Apart from gut, Talbot (1977) show that *S. noctilio* salivary secretions putatively played an important role in the digestion of fungi. Above mentioned, further research is required to confirm this observation.

### Contribution of Gut Bacteria and Fungi to *S. noctilio* Larval Nutrition

Given the limited digestion capacity of the gut, gut microbiota may play an important role in overcoming nutritional deficiencies. In this study, we first used both 16S rRNA and fungal ITS2 region to explore the larval and adult guts and larval frass to reveal the features of the microbial community of *S. noctilio.*

Bacterial communities in *S. noctilio* gut and frass varied significantly, whereas there was no significant difference in fungal communities. The term frass commonly refers to insect feces. Still, for *Sirex*, frass refers to chewed xylem that mixes with a small amount of excrement from the trailing gallery left by foraging larva (Thompson, 2013). Hence, frass was also included in our study to compare with the insect’s gut microbiota. We found that the gut microbial communities were more similar between the female and male adults than that of larvae, indicating that the host developmental stage could influence the microbial community. This result was consistent with studies on Lepidopteran holometabolous insects (Wang et al., 2020). The gut bacterial and fungal community structures and diversity were similar between female and male adults of *S. noctilio*, which was in agreement with previous findings in *Laodelphax striatellus*, although Hemiptera is hemimetabolous insect (Bing et al., 2020). Under the unweighted Unifrac, the bacterial and fungal communities clustered further apart, indicating that there might be more unique species in each group. Under the weighted Unifrac, the distance between each group of samples seemed closer, indicating that the relative abundance of their unique species might be lower, while the relative abundance of the common species was closer. Since bacterial populations severely disrupt or significantly decrease in pupal stage and recover only 4–6 d after adult emergence (Hakim et al., 2010; Hroncova et al., 2015), we did not study *S. noctilio* gut at the pupal stage.

Bacterial communities in *S. noctilio* larval gut were dominated by *Pseudomonas*, followed by *Ralstonia*, *Rhodococcus*, *Enhydrobacter*, *Bosea*, *Curvibacter*, and *Bradyrhizobium*. These typical taxa include bacteria from the phyla Proteobacteria and Actinobacteria, and some of the former in other insects seem to supplement nutrients and are necessary for normal growth (Watanabe et al., 2003). In addition, *Pseudomonas* species can grow at a wide range of temperatures from 5 to 42°C (Termine and Michel, 2009), which might be beneficial for larvae to adapt better to the environment. Most of the OTUs were conserved across both larvae and adults, such as *Ralstonia*, *Pseudomonas*, *Burkholderia* s.l, *Stenotrophomonas*, *Acinetobacter*, *Curvibacte*r, *Enhydrobacter*, *Methylobacterium*, etc., indicating that these bacterial genera were the resident gut microbiota and may be functionally relevant for the host. Intriguingly, the relative abundance of these core bacterial genera was different between larvae and adults’ guts. This may partly be due to the adult woodwasp’s not feeding on the host tree but stored fat-body reserves (Taylor, 1981). Our results also reflected the slight difference of the gut microbial communities and functional predictions between guts and frass (Supplementary Figure 7). *Zoogloea*, *Ruminobacter*, *Nitrospira*, and *Nitrosospira* were found only in larval gut samples, which were reported to be efficient in the degradation of organic matter and play a role in the nitrogen cycle (Anderson, 1995; Daims et al., 2015). These findings suggest gut microbiota fluctuations in *S. noctilio* when they feed on recalcitrant food sources. Adams et al. (2011) report that *Streptomyces* and *Pantoea* present in *S. noctilio* could degrade cellulose and assist in nutrient intake, but these two genera were not observed in our study, implying that these may be from other organs of the insect body, or their presence may vary across geographic regions.

Fungal communities in *S. noctilio* larval gut were dominated by *Amylostereum*. Other core OTUs were conserved across both larvae and adults, such as *Trichoderma*, *Hyphopichia*, etc. The unique fungal genera in larval guts were mainly *Pichia* and *Ceratocystis*, also the wood rotting fungi. Similarly, yeast was found in the guts of *Apis* spp., the carpenter bee *Xylocopa* spp., and Vespidae (Stefanini, 2018). The obligate mutualist *A. areolatum* is beneficial to the larvae, while the facultative symbionts may assist their insect hosts in digestion and xenobiotic detoxification (Gupta and Nair, 2020). The development of woodwasp larvae is correlated with the growth of the symbiotic fungus *Amylostereum*; larvae feed exclusively on the fungus until the third instar and then on fungus-colonized wood (preferentially at the border of fungal growth in wood) (Taylor, 1981; Thompson et al., 2014). In our findings, the high relative abundance of this fungus in the gut was probably due to its roles involved with food sources. Furthermore, we observed very few fungal OTUs in our dataset, which could be due to the primer selection or database imperfection leading to the unclassified taxa. Further studies are required for in-depth analysis of the fungal OTUs.

Bacterial-fungal interactions existing in insects are very important. Various microbes such as bacteria, archaea, and fungi interact with each other in their respective insect hosts (Gurung et al., 2019). The bacterial communities changed greatly with the growth and development of *S. noctilio*, while the fungal communities were more stable. Our results showed that some bacteria and fungi might be positively or negatively correlated in *S. noctilio* larval gut. Our findings suggested the importance of evaluating microbial diversity in insects that share a similar ecological niche, and some bacteria may have mutually beneficial interactions with fungi, same as reported by Bing et al. (2020).

The duramen (or heartwood) is mainly made up of cellulose, hemicellulose, and lignin. Different digestive enzymes are required for different substrates, which may sometimes act in congruence (Chiappini and Aldini, 2011). Some species feed on predegraded wood (Colepicolo-Neto et al., 1986), similarly, although the symbiotic fungus *A. areolatum* may help in primary degradation (Fu et al., 2020), gut microbiota also contributes to these processes (Schloss et al., 2006; Le Roes-Hill et al., 2011). We predicted the presence of several genes encoding enzymes in gut microbiota involved in degradation: several peroxidases, oxidizing phenolic/non-phenolic compounds and modifying lignin polymers (Levasseur et al., 2013); glycolate oxidase, oxidizing glycolate to glyoxylate and producing reactive oxygen species; and gluco-oligosaccharide oxidases, oxidizing different carbohydrates, which may be involved in the lignocellulose degradation (van Hellemond et al., 2006). For degradation of cellulose and hemicellulose, genes encode enzymes such as endoglucanase, cleaving internal bonds in cellulose (Klyosov, 1990); beta-glucosidase, hydrolyzing cellobiose and short-chain oligosaccharides (Glass et al., 2013). Our results showed that genes that encode carboxylesterase and beta-glucosidase were predicted in the gut fungal communities. Carboxylesterase also shows insecticide resistance in *Myzus persicae* (Devonshire and Moores, 1982). We also predicted genes that encode cellobiosidase, alpha-L-fucosidase, endo1,4-b-xylanase, and alpha-N-arabinofuranosidase [(hemi)cellulosic accessory enzymes catalyzing the hydrolysis of arabinans, arabinoxylans, alpha-l-fucosyl residues (Numan and Bhosle, 2006)]; xylan1,4-beta-xylosidase [glycosidase hydrolyzing linkage between beta-linked xylose residues in beta-1,4 xylan (Zhou et al., 2012)]; beta-galactosidase [enzyme hydrolyzing beta-galactosidic bonds (Husain, 2010)]; and beta-mannosidase (enzyme hydrolyzing terminal beta-H-mannose residues in beta-D-mannosides). Most of the genes predicted in our study are also detected in several cerambycid larvae such as *Anoplophora glabripennis*, *Trichoferus campestris*, etc. (Scully et al., 2013; Mohammed et al., 2018).

For nitrogen fixation, the key genes (*nifH*, *nifD*, and *nifK* for nitrogenase component proteins) (Gaby and Buckley, 2014), were predicted in larval gut bacterial communities. These genes might be involved in nitrogen fixation and provide nitrogen to insects (Ramírez-Puebla et al., 2013). Similar with our results, *Sirex* frass was enriched with nitrogen compared to pine xylem (Thompson, 2013), indicating nitrogen fixation by larval gut bacteria.

Both structure and microbiota of the gut may help providing physiological and molecular adaptations to their woodwasp hosts. The gut microbiota may also facilitate the survival of other microbes in other body parts of the woodwasp, or mediate indirect metabolic interactions. In-depth understanding of the gut structure and its microbiota can help in *S. noctilio* pest control by developing strategies for interfering with the gut microbiota. Further research is required to understand the relationship between the composition of the gut microbiota of the *S. noctilio* and its geographic distribution, and its similarities and differences with the local species *S. nitobei*. The co-evolution of the mutualism among *S. noctilio*, its symbiotic fungus *A. areolatum*, and its gut microbiota also needs to be looked into.

## Conclusion

Our characterization of the *S. noctilio* gut structure and gut microbiota suggest their role in the survival of the larvae in nutrient-deficient host xylem. The larval gut was thin and thread-like from the oral cavity to the anus. It lacked distinct fermentation chambers, which indicates its limited, but not ineffective, capacity to digest xylem. A wide variation in bacterial communities was observed in guts of larvae and adults, and larval frass, and fungal communities did not change significantly in different developmental stages and frass. *Pseudomonas*, *Ralstonia*, and *Burkholderia* s.l were dominant in the guts of larvae, adults, and frass, respectively, *Amylostereum* was dominant in *S. noctilio* larval gut. Correlation analysis showed that the bacteria and fungi with higher abundance in the larval gut also exhibited varying degrees of positive or negative correlation. Functional predictions of bacterial and fungal communities inhabiting the larval gut suggest a role in degrading lignocellulose and fixing nitrogen. Therefore, we hypothesized that *Sirex* larvae may rely on the symbiotic fungus *Amylostereum* for lignin digestion and food resources; the gut bacteria may play an important role in fixing nitrogen and degrading lignocellulose for the survival of *Sirex* larvae.

## Data Availability Statement

The raw Illumina sequencing data for the 16S rRNA and ITS2 sequences can be found in the NCBI SRA database under GenBank accession numbers PRJNA683715.

## Ethics Statement

All samples were collected from pine plantations invaded with *Sirex noctilio*, and permission was not required. Tested state-owned forest farms did not involve any endangered or protected species, and experimental plots did not have private ownership issues. Efforts were made to minimize suffering of species during sample collection.

## Author Contributions

JL and LR contributed to the design of the study. JL, CL, LW, and MW collected the samples in all period. JL and CG collected gut content samples. JL and XL observed the gut structure. JL analyzed the data. JL, LR, and YL contributed to the writing and editing of the manuscript. All authors read and approved the final manuscript.

## Conflict of Interest

The authors declare that the research was conducted in the absence of any commercial or financial relationships that could be construed as a potential conflict of interest.

## Footnotes

1 http://drive5.com/uparse/

2 http://sourceforge.net/projects/rdp-classifier/

3 http://mothur.org/wiki/miseq_sop/#alpha-diversity-1
