# Supplementary material for: Gut Structure and Microbial Communities in Sirex noctilio (Hymenoptera: Siricidae) and Their Predicted Contribution to Larval Nutrition
Source: Front Microbiol. 2021 Apr 8;12:641141. doi: 10.3389/fmicb.2021.641141 (PMC8060704; doi:10.3389/fmicb.2021.641141)
Supplement: Supplementary file 1 [file Data_Sheet_1.PDF]

Supplementary Material

1 Supplementary Figures and Tables

1.1 Supplementary Figures

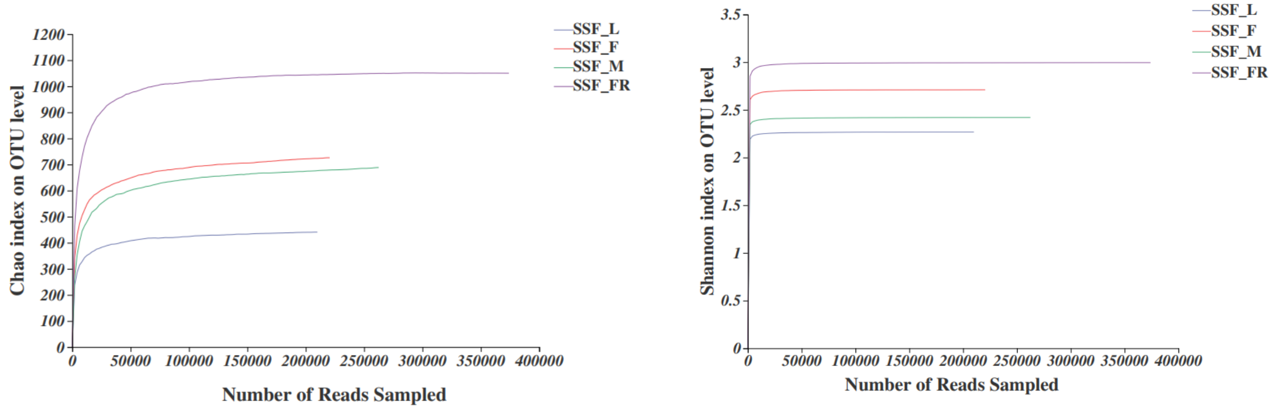

Figure S1. Rarefaction curves of gut and frass bacterial communities.
(A) Chao index and **(B)** Shannon index. Each line represents a sampling period, and each period is the mean ± SE. (SSF_L, larva, gray blue, *n* = 25; SSF_F, female, red, *n* = 30; SSF_M, male, green, *n* = 35; SSF_FR, frass, purple, *n* = 30). Rarefaction plots were produced in Mothur (v.1.30.1 http://www.mothur.org/wiki/Schloss_SOP#Alpha_diversity) and R v3.6.1.

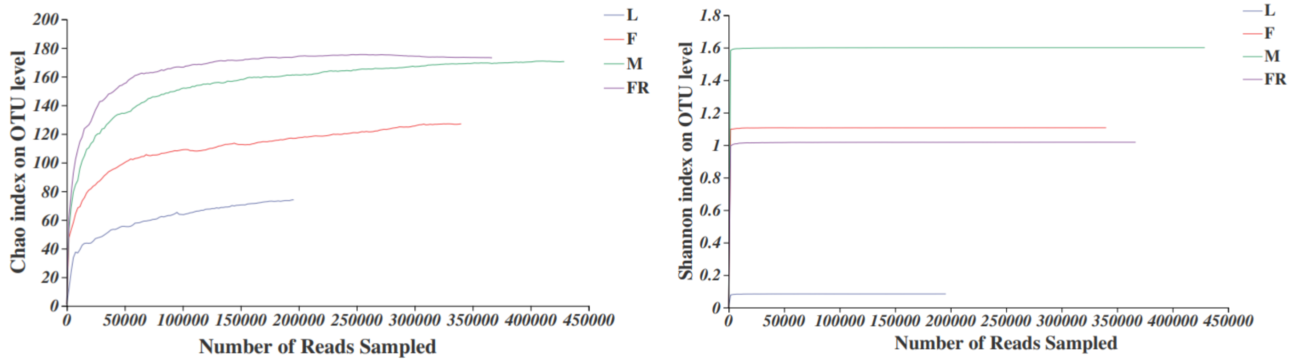

Figure S2. Rarefaction curves of gut and frass fungal communities.
(A) Chao index and **(B)** Shannon index. Each line represents a sampling period, and each period is the mean ± SE. (L, larva, gray blue, *n* = 25; F, female, red, *n* = 30; M, male, green, *n* = 35; FR, frass, purple, *n* = 30). Rarefaction plots were produced in Mothur (v.1.30.1 http://www.mothur.org/wiki/Schloss_SOP#Alpha_diversity) and R v3.6.1.

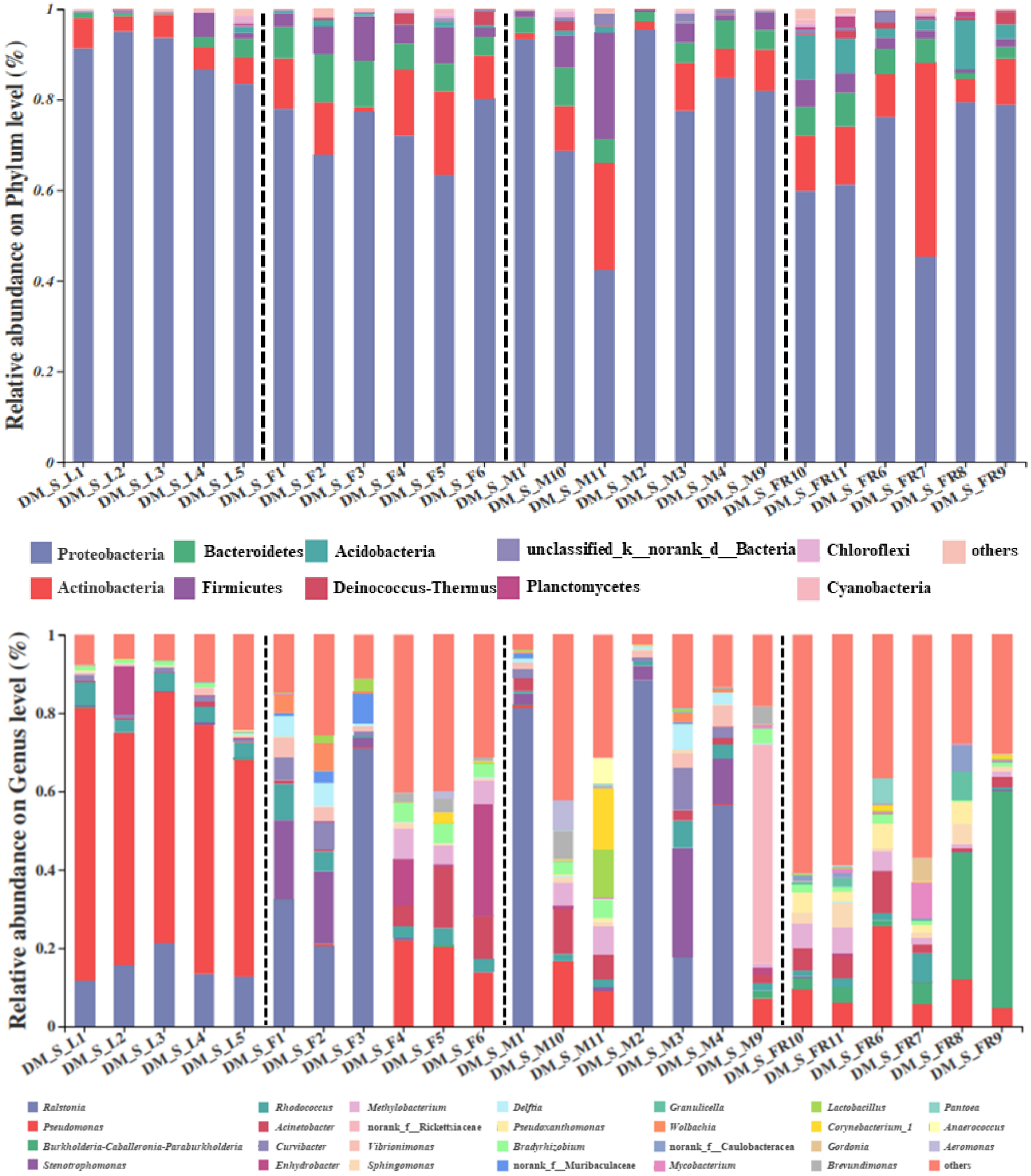

Figure S3. Relative abundance of bacterial communities associated with *Sirex noctilio* gut and frass.

(A) At the phylum level. Each bar is indicated by a different color at the phylum level. OTUs that were 1% of average relative abundance in groups are summarized as “others”. **(B)** At the genus level. Each bar is indicated by a different color at the genus level. OTUs that were < 5% of average relative abundance in groups are summarized as “others.” (DM_S_L, larva; DM_S_F, female; DM_S_M, male; DM_S_FR, frass.)

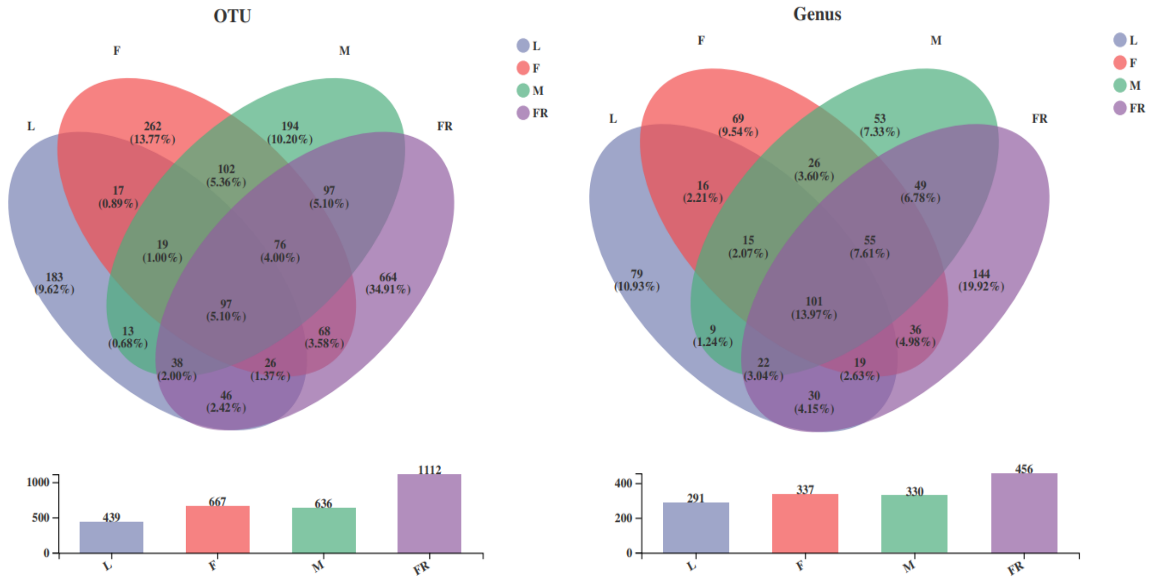

Figure S4. Venn diagrams depicting the overlap of the bacterial community associated with *Sirex noctilio* gut and frass.
(A) At OTU level and **(B)** at the genus level. (L, larva; F, female; M, male; FR, frass.)

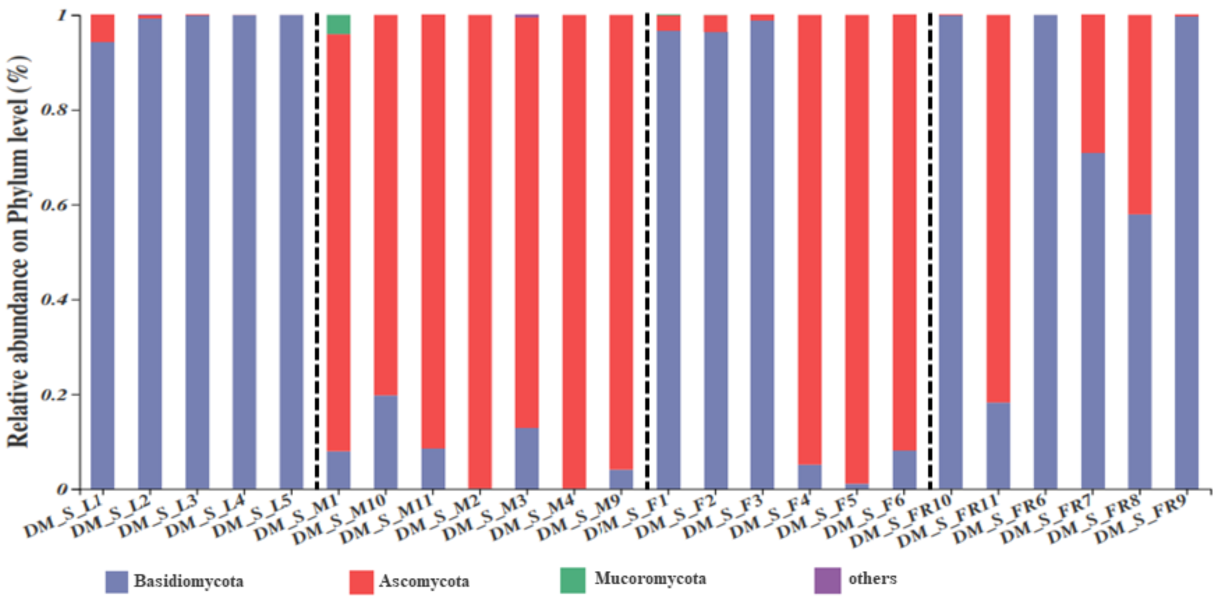

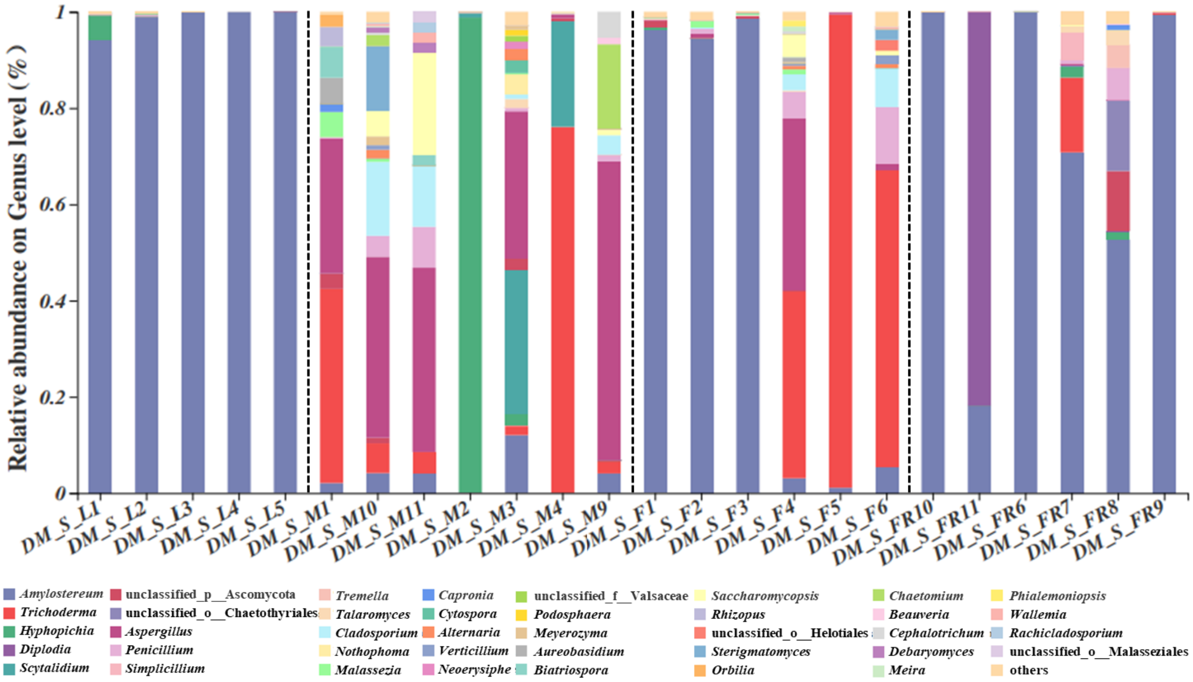

Figure S5. Relative abundance of fungal communities associated with *Sirex noctilio* gut and frass.
(A) At the phylum level. Each bar is indicated by a different color at the phylum level. OTUs that were 1% of average relative abundance in groups are summarized as “others”. **(B)** At the genus level. Each bar is indicated by a different color at the genus level. OTUs that were < 1% of average relative abundance in groups are summarized as “others”. (DM_S_L, larva; DM_S_F, female; DM_S_M, male; DM_S_FR, frass.)

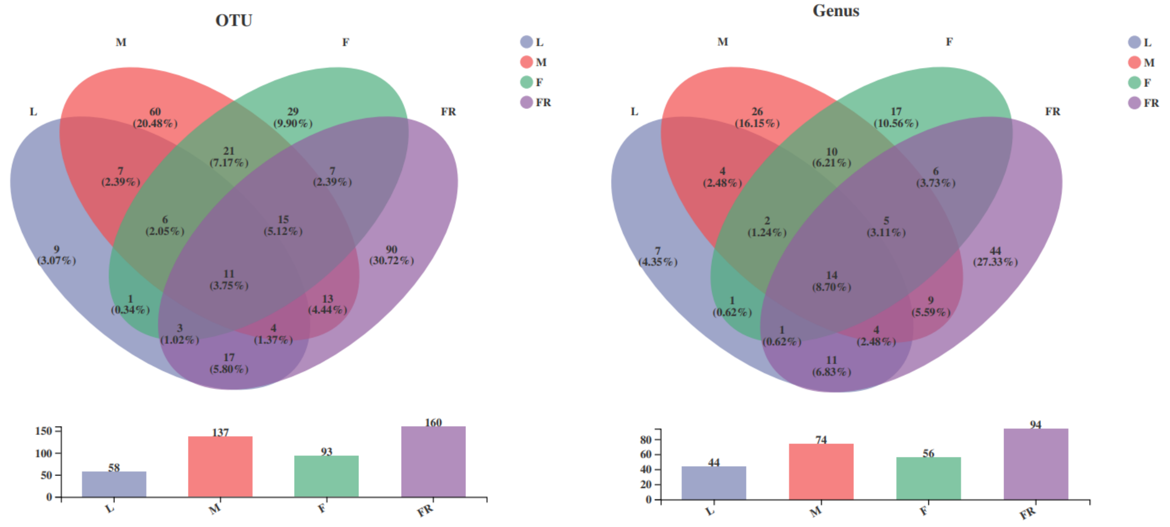

Figure S6. Venn diagrams depicting the overlap of the fungal community associated with *Sirex noctilio* gut and frass.
(A) At OTU level and **(B)** at the genus level. (L, larva; F, female; M, male; FR, frass.)

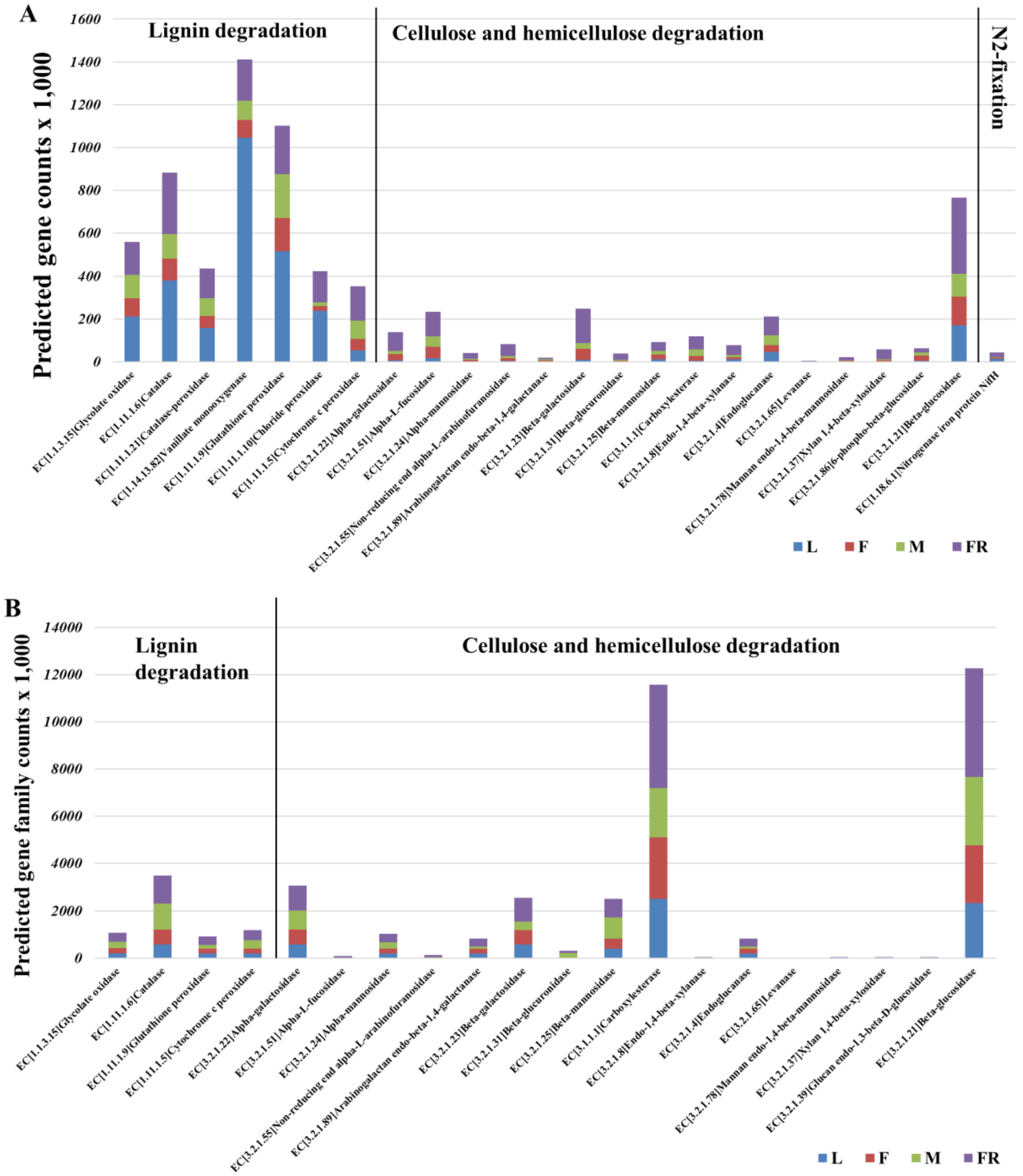

Figure S7. Selection of genes involved in the lignocellulose degradation and nitrogen fixation of *Sirex noctilio* larva, adult gut and larval frass.
(A) Bacterial and **(B)** fungal communities (the sum number of predicted gene family counts). (L, larva; F, female; M, male; FR, frass.)

1. 2 Supplementary Tables

Table S1. Primer sequences used in this study

Primer name	Primer sequence (5'-3')	length
LCO1490	GGTCAACAAATCATAAAGATATTGG	700bp
HCO2198	TAAACTTCAGGGTGACCAAAAAATCA	
16S-338F	ACTCCTACGGGAGGCAGCAG	468bp
16S-806R	GGACTACHVGGGTWTCTAAT	
ITS3F	GCATCGATGAAGAACGCAGC	200~500bp
ITS4R	TCCTCCGCTTATTGATATGC	

Table S2. Summary statistics for the Illumina MiSeq runs for all samples

Sample ID	16S rRNA gene				ITS region			
	Mean length	Number of received reads	Number of filtered reads		Mean length	Number of received reads	Number of filtered reads	
DM_S_L1	426	54005	53116	(98%)	325	33654	33593	(100%)
DM_S_L2	426	45703	44723	(98%)	330	31400	31349	(100%)
DM_S_L3	427	39975	39204	(98%)	330	44362	44288	(100%)
DM_S_L4	425	28524	27486	(96%)	330	40627	40577	(100%)
DM_S_L5	394	45330	43193	(95%)	330	44736	44680	(100%)
DM_S_F1	424	28722	26880	(94%)	314	107961	81368	(75%)
DM_S_F2	422	27463	25468	(93%)	330	41273	41094	(100%)
DM_S_F3	426	43274	40137	(93%)	331	80325	79379	(99%)
DM_S_F4	410	36532	11958	(33%)	455	34583	392	(1%)
DM_S_F5	408	41138	6817	(17%)	448	42365	2858	(7%)
DM_S_F6	409	47854	7124	(15%)	456	36602	222	(1%)
DM_S_M1	428	46347	45152	(97%)	327	1405	1210	(86%)
DM_S_M10	408	51361	6080	(12%)	456	47032	436	(1%)
DM_S_M11	408	44048	7591	(17%)	457	39939	47	(0%)
DM_S_M2	428	47770	46628	(98%)	243	73974	73917	(100%)
DM_S_M3	424	25771	24587	(95%)	317	15143	13842	(91%)
DM_S_M4	426	19356	18142	(94%)	320	40857	40611	(99%)
DM_S_M9	408	31117	12945	(42%)	457	43791	74	(0%)
DM_S_FR10	409	69932	20365	(29%)	330	65923	65826	(100%)
DM_S_FR11	409	68187	23949	(35%)	310	59478	59444	(100%)
DM_S_FR6	408	61243	9137	(15%)	330	65303	65211	(100%)
DM_S_FR7	411	57639	34779	(60%)	325	55310	54684	(99%)
DM_S_FR8	417	64232	51589	(80%)	321	50963	50359	(99%)
DM_S_FR9	416	63442	36444	(57%)	330	69994	69895	(100%)

Sample ID	16S rRNA gene				ITS region			
	Mean length	Number of received reads	Number of filtered reads		Mean length	Number of received reads	Number of filtered reads	
DM_S_L	420	213537	207722	97.28%	329	194779	194487	99.85%
DM _S_F	417	224983	118384	52.60%	389	343109	205310	59.84%

DM_S_M	419	265770	161125	60.63%	368	262141	130137	49.64%
DM_S_FR	412	384675	176263	45.82%	324	366971	365419	99.58%

Table S3. Summary of observed bacterial and fungal OTUs, Shannon, Simpson, ACE, Chao and coverage

Sample ID	bacteria						fungi					
	OTUs	Shannon	Simpson	ACE	Chao	Coverage	OTUs	Shannon	Simpson	ACE	Chao	Coverage
DM_S_L1	148	1.96	0.31	159.04	186.25	1.00	32	0.27	0.89	36.62	37.25	1.00
DM_S_L2	133	2.02	0.25	141.98	145.00	1.00	36	0.09	0.98	39.54	43.00	1.00
DM_S_L3	103	1.84	0.29	110.94	118.60	1.00	5	0.00	1.00	11.00	6.50	1.00
DM_S_L4	80	2.08	0.26	84.80	85.60	1.00	13	0.01	1.00	27.81	27.00	1.00
DM_S_L5	229	2.81	0.20	239.06	244.00	1.00	6	0.00	1.00	16.00	9.00	1.00
DM_S_F1	116	2.64	0.12	128.12	133.14	1.00	26	0.27	0.92	28.72	29.00	1.00
DM_S_F2	151	3.23	0.08	161.37	161.50	1.00	29	0.36	0.89	29.98	30.00	1.00
DM_S_F3	290	2.32	0.29	299.81	309.46	1.00	31	0.10	0.97	32.11	31.25	1.00
DM_S_F4	172	3.51	0.07	182.06	184.36	1.00	33	2.54	0.12	39.48	40.20	0.98
DM_S_F5	162	3.74	0.06	168.58	175.13	1.00	8	0.10	0.97	21.78	9.00	1.00
DM_S_F6	130	3.02	0.12	149.53	155.00	1.00	19	1.71	0.35	22.45	19.86	0.98
DM _S_M1	179	1.60	0.39	190.24	196.77	1.00	18	1.93	0.24	18.41	18.00	1.00
DM_S_M10	196	3.78	0.05	206.93	211.81	1.00	39	2.70	0.12	42.95	42.00	0.98
DM_S_M11	187	3.75	0.05	200.46	200.32	1.00	21	2.80	0.05	34.55	32.00	0.77
DM_S_M2	114	1.27	0.39	122.49	119.71	1.00	29	0.09	0.97	33.25	36.50	1.00
DM_S_M3	158	2.85	0.13	170.34	183.50	1.00	45	2.48	0.16	45.54	45.00	1.00
DM_S_M4	160	2.35	0.20	178.60	185.38	1.00	35	0.67	0.63	37.90	38.33	1.00
DM_S_M9	123	2.27	0.32	197.49	189.43	1.00	12	1.99	0.16	21.46	15.00	0.95
DM_S_FR10	376	4.75	0.02	389.63	389.04	1.00	12	0.00	1.00	47.89	26.00	1.00
DM_S_FR11	487	5.08	0.01	510.36	521.73	1.00	5	0.48	0.70	22.13	8.00	1.00
DM_S_FR6	234	4.02	0.04	240.69	241.65	1.00	5	0.00	1.00	12.60	8.00	1.00
DM_S_FR7	437	4.62	0.02	448.95	453.71	1.00	115	1.17	0.53	118.33	117.55	1.00
DM_S_FR8	439	3.56	0.10	469.14	492.13	1.00	104	1.69	0.32	107.32	106.33	1.00
DM_S_FR9	397	2.94	0.30	420.50	432.45	1.00	43	0.04	0.99	103.06	66.10	1.00

Table S4. Taxonomic information of bacterial and fungal communities in different groups

Sample		Number of OTUs	Number of different taxonomic categories					
			Phylum	Class	Order	Family	Genus	Species
Bacteria	DM_S_L	415	22	42	111	176	295	363
	DM_S_F	624	24	47	108	180	336	452
	DM_S_M	592	19	35	87	158	335	466
	DM_S_FR	964	24	42	112	217	452	677
Fungi	DM_S_L	58	3	14	24	37	44	54
	DM_S_F	92	3	16	31	50	57	74
	DM_S_M	115	4	19	35	51	62	85
	DM_S_FR	160	2	16	41	74	94	123

Table S5. A) Results of Unweighted UniFrac PERMANOVA analysis for bacterial communities

	Df	SumsOfSqs	MeanSqs	F.Model	R2	Pr(>F)
group_factor$DM	3	1.847823376	0.615941125	2.438410314	0.267807765	0.001***
Residuals	20	5.051989173	0.252599459	-	0.732192235	-
Total	23	6.899812549	-	-	1	-

Table S5. B) Results of weighted UniFrac PERMANOVA analysis for bacterial communities

	Df	SumsOfSqs	MeanSqs	F.Model	R2	Pr(>F)
group_factor$DM	3	0.465456965	0.155152322	2.882105008	0.301829922	0.002**
Residuals	20	1.076659741	0.053832987	-	0.698170078	-
Total	23	1.542116706	-	-	1	-

Table S6. Bacterial taxa in *Sirex noctilio* gut and frass

Phylum	Class	Order	Family	Genus
Proteobacteria	Gammaproteobacteria	Betaproteobacteriales	Burkholderiaceae	*Ralstonia*
	Gammaproteobacteria	Pseudomonadales	Pseudomonadaceae	*Pseudomonas*
	Gammaproteobacteria	Betaproteobacteriales	Burkholderiaceae	*Burkholderia-Caballeronia-Paraburkholderia*
	Gammaproteobacteria	Xanthomonadales	Xanthomonadaceae	*Stenotrophomonas*
	Gammaproteobacteria	Pseudomonadales	Moraxellaceae	*Acinetobacter*
	Gammaproteobacteria	Betaproteobacteriales	Burkholderiaceae	*Curvibacter*
	Gammaproteobacteria	Pseudomonadales	Moraxellaceae	*Enhydrobacter*
	Alphaproteobacteria	Rhizobiales	Beijerinckiaceae	*Methylobacterium*
	Alphaproteobacteria	Rickettsiales	Rickettsiaceae	*
	Alphaproteobacteria	Sphingomonadales	Sphingomonadaceae	*Sphingomonas*
	Gammaproteobacteria	Betaproteobacteriales	Burkholderiaceae	*Delftia*
	Gammaproteobacteria	Xanthomonadales	Xanthomonadaceae	*Pseudoxanthomonas*
	Alphaproteobacteria	Rhizobiales	Xanthobacteraceae	*Bradyrhizobium*
	Gammaproteobacteria	Betaproteobacteriales	Burkholderiaceae	*Noviherbaspirillum*
	Alphaproteobacteria	Rhizobiales	Beijerinckiaceae	*Bosea*
	Alphaproteobacteria	Rickettsiales	Anaplasmataceae	*Wolbachia*
Actinobacteria	Actinobacteria	Corynebacteriales	Nocardiaceae	*Rhodococcus*
	Actinobacteria	Corynebacteriales	Mycobacteriaceae	*Mycobacterium*
	Actinobacteria	Micrococcales	Microbacteriaceae	*Microbacterium*
	Actinobacteria	Micrococcales	Microbacteriaceae	*Leifsonia*
	Actinobacteria	Corynebacteriales	Corynebacteriaceae	*
Bacteroidetes	Bacteroidia	Chitinophagales	Chitinophagaceae	*Vibrionimonas*
	Bacteroidia	Bacteroidales	Muribaculaceae	*
	Bacteroidia	Chitinophagales	Chitinophagaceae	*Hydrotalea*
Firmicutes	Bacilli	Lactobacillales	Lactobacillaceae	*Lactobacillus*
	Bacilli	Bacillales	Staphylococcaceae	*Staphylococcus*
	Bacilli	Clostridiales	Clostridiaceae	*
Acidobacteria	Acidobacteriia	Acidobacteriales	Acidobacteriaceae	*Granulicella*
	Acidobacteriia	Solibacterales	Solibacteraceae	*Bryobacter*
Deinococcus-Thermus	Deinococci	Deinococcales	Deinococcaceae	*Deinococcus*

Asterisks indicate unclassified members of higher level taxa; and the red font indicates the common bacterial genera of the four groups.

Table S7. A) Results of Unweighted UniFrac PERMANOVA analysis for fungal communities

	Df	SumsOfSqs	MeanSqs	F.Model	R2	Pr(>F)

group_factor$DM	3	1.203768933	0.401256311	1.587233862	0.171520523	0.01*
Residuals	20	5.814452032	0.252802262	-	0.828479477	-
Total	23	7.018220966	-	-	1	-

B) Results of weighted UniFrac PERMANOVA analysis for fungal communities

	Df	SumsOfSqs	MeanSqs	F.Model	R2	Pr(>F)
group_factor$DM	3	4.777874391	1.592624797	4.2166884	0.35483989	0.005**
Residuals	20	8.686999572	0.377695634	-	0.64516011	-
Total	23	13.46487396	-	-	1	-

Table S8. Fungal taxa in *Sirex noctilio* gut and frass

Phylum	Class	Order	Family	Genus
Basidiomycota	Agaricomycetes	Russulales	Stereaceae	*Amylostereum*
	Tremellomycetes	Tremellales	Tremellaceae	*Tremella*
	Malasseziomycetes	Malasseziales	Malasseziaceae	*Malassezia*
Ascomycota	Sordariomycetes	Hypocreales	Hypocreaceae	*Trichoderma*
	Saccharomycetes	Saccharomycetales	Debaryomycetaceae	*Hyphopichia*
	Dothideomycetes	Botryosphaeriales	Botryosphaeriaceae	*Diplodia*
	Leotiomycetes	Helotiales	Helotiaceae	*Scytalidium*
	Eurotiomycetes	Chaetothyriales	*	*
	Eurotiomycetes	Eurotiales	Aspergillaceae	*Aspergillus*
	Eurotiomycetes	Eurotiales	Aspergillaceae	*Penicillium*
	Sordariomycetes	Hypocreales	Cordycipitaceae	*Simplicillium*
	Dothideomycetes	Capnodiales	Cladosporiaceae	*Cladosporium*

Asterisks indicate unclassified members of higher level taxa; and the red font indicates the common fungal genera of the four groups.

Table S9. Selection of bacterial genes involved in lignocellulose metabolism and nitrogen fixation (with the PICRUSt2 software) as well as the enzyme-catalyzed reactions

	[EC number]	KEGG gene description	Reaction (IUBMB)
**Lignin**	1.1.3.15	Glycolate oxidase	an (S)-2-hydroxy carboxylate + O2 = a 2-oxo carboxylate + H2O2
	1.11.1.6	Catalase	2 H2O2 = O2 + 2 H2O
	1.11.1.21	Catalase-peroxidase	(1) donor + H2O2 = oxidized donor + 2 H2O; (2) 2 H2O2 = O2 + 2 H2O
	1.14.13.82	Vanillate monooxygenase	vanillate + O2 + NADH + H+ = 3,4-dihydroxybenzoate + NAD+ + H2O + formaldehyde
	1.11.1.9	Glutathione peroxidase	2 glutathione + H2O2 = glutathione disulfide + 2 H2O
	1.11.1.10	Chloride peroxidase	RH + chloride + H2O2 = RCl + 2 H2O
	1.11.1.5	Cytochrome c peroxidase	2 ferrocytochrome c + H2O2 = 2 ferricytochrome c + 2 H2O
**Cellulose and hemicellulose**	3.2.1.22	Alpha-galactosidase	hydrolysis of terminal, non-reducing alpha-D-galactose residues in alpha-Dgalactosides, including galactose oligosaccharides, galactomannans and galactolipids
	3.2.1.51	Alpha-L-fucosidase	an alpha-L-fucoside + H2O = L-fucose + an alcohol
	3.2.1.24	Alpha-mannosidase	hydrolysis of terminal, non-reducing alpha-D-mannose residues in alpha-D mannosides

	3.2.1.55	Non-reducing end alpha-L-arabinofuranosidase	hydrolysis of terminal non-reducing alpha-L-arabinofuranoside residues in alpha-Larabinosides
	3.2.1.89	Arabinogalactan endo-beta-1,4-galactanase	the enzyme specifically hydrolyses (1->4)-beta-D-galactosidic linkages in type I arabinogalactans
	3.2.1.23	Beta-galactosidase	hydrolysis of terminal non-reducing beta-D-galactose residues in beta-D galactosides
	3.2.1.31	Beta-glucuronidase	a beta-D-glucuronoside + H2O = D-glucuronate + an alcohol
	3.2.1.25	Beta-mannosidase	hydrolysis of terminal, non-reducing beta-D-mannose residues in beta-D mannosides
	3.1.1.1	Carboxylesterase	a carboxylic ester + H2O = an alcohol + a carboxylate
	3.2.1.8	Endo-1,4-beta-xylanase	endohydrolysis of (1->4)-beta-D-xylosidic linkages in xylans
	3.2.1.4	Endoglucanase	endohydrolysis of (1->4)-beta-D-glucosidic linkages in cellulose, lichenin andcereal beta-D-glucans
	3.2.1.65	Levanase	random hydrolysis of (2->6)-beta-D-fructofuranosidic linkages in (2->6)-beta-Dfructans (levans) containing more than 3 fructose units
	3.2.1.78	Mannan endo-1,4-beta-mannosidase	random hydrolysis of (1->4)-beta-D-mannosidic linkages in mannans, galactomannans and glucomannans
	3.2.1.37	Xylan 1,4-beta-xylosidase	hydrolysis of (1->4)-beta-D-xylans, to remove successive D-xylose residues fromthe non-reducing termini
	3.2.1.86	6-phospho-beta-glucosidase	6-phospho-beta-D-glucosyl-(1->4)-D-glucose + H2O = D-glucose + D-glucose 6-phosphate
	3.2.1.21	Beta-glucosidase	hydrolysis of terminal, non-reducing beta-D-glucosyl residues with release of betaD-glucose
**N2-fixation**	1.18.6.1	Nitrogenase iron protein NifH	8 reduced ferredoxin + 8 H+ + N2 + 16 ATP + 16 H2O = 8 oxidized ferredoxin + H2+ 2 NH3 + 16 ADP + 16 phosphate

Table S10. Selection of fungal genes involved in lignocellulose metabolism and nitrogen fixation (with the PICRUSt2 software) as well as the enzyme-catalyzed reactions

	**[EC number]**	**KEGG gene description**	**Reaction (IUBMB)**
**Lignin**	1.1.3.15	Glycolate oxidase	an (S)-2-hydroxy carboxylate + O2 = a 2-oxo carboxylate + H2O2
	1.11.1.6	Catalase	2 H2O2 = O2 + 2 H2O
	1.11.1.9	Glutathione peroxidase	2 glutathione + H2O2 = glutathione disulfide + 2 H2O
	1.11.1.5	Cytochrome c peroxidase	2 ferrocytochrome c + H2O2 = 2 ferricytochrome c + 2 H2O
**Cellulose and hemicellulose**	3.2.1.22	Alpha-galactosidase	hydrolysis of terminal, non-reducing alpha-D-galactose residues in alpha-Dgalactosides, including galactose oligosaccharides, galactomannans and galactolipids
	3.2.1.51	Alpha-L-fucosidase	an alpha-L-fucoside + H2O = L-fucose + an alcohol
	3.2.1.24	Alpha-mannosidase	hydrolysis of terminal, non-reducing alpha-D-mannose residues in alpha-D mannosides
	3.2.1.55	Non-reducing end alpha-L-arabinofuranosidase	hydrolysis of terminal non-reducing alpha-L-arabinofuranoside residues in alpha-Larabinosides
	3.2.1.89	Arabinogalactan endo-beta-1,4-galactanase	the enzyme specifically hydrolyses (1->4)-beta-D-galactosidic linkages in type I arabinogalactans

3.2.1.23	Beta-galactosidase	hydrolysis of terminal non-reducing beta-D-galactose residues in beta-D galactosides
3.2.1.31	Beta-glucuronidase	a beta-D-glucuronoside + H2O = D-glucuronate + an alcohol
3.2.1.25	Beta-mannosidase	hydrolysis of terminal, non-reducing beta-D-mannose residues in beta-D mannosides
3.1.1.1	Carboxylesterase	a carboxylic ester + H2O = an alcohol + a carboxylate
3.2.1.8	Endo-1,4-beta-xylanase	endohydrolysis of (1->4)-beta-D-xylosidic linkages in xylans
3.2.1.4	Endoglucanase	endohydrolysis of (1->4)-beta-D-glucosidic linkages in cellulose, lichenin andcereal beta-D-glucans
3.2.1.65	Levanase	random hydrolysis of (2->6)-beta-D-fructofuranosidic linkages in (2->6)-beta-Dfructans (levans) containing more than 3 fructose units
3.2.1.78	Mannan endo-1,4-beta-mannosidase	random hydrolysis of (1->4)-beta-D-mannosidic linkages in mannans, galactomannans and glucomannans
3.2.1.37	Xylan 1,4-beta-xylosidase	hydrolysis of (1->4)-beta-D-xylans, to remove successive D-xylose residues fromthe non-reducing termini
3.2.1.39	Glucan endo-1,3-beta-D-glucosidase	hydrolysis of (1->3)-beta-D-glucosidic linkages in (1->3)-beta-D-glucans
3.2.1.21	Beta-glucosidase	hydrolysis of terminal, non-reducing beta-D-glucosyl residues with release of betaD-glucose
